# Specialists and generalists coexist within a population of spider-hunting mud dauber wasps

**DOI:** 10.1093/beheco/arx050

**Published:** 2017-04-01

**Authors:** Erin C. Powell, Lisa A. Taylor

**Affiliations:** a Entomology and Nematology Department, University of Florida, 1881 Natural Area Drive, Gainesville, FL 32611, USA,; b School of Biological Sciences, University of Auckland, 3A Symonds St, Auckland Central 1010, New Zealand, and; c Florida Museum of Natural History, University of Florida, 3215 Hull Road, Gainesville, FL 32611, USA

**Keywords:** Araneae, individual differences, individual specialization, predator psychology, search images, Sphecidae.

## Abstract

Individual foraging specialization describes the phenomenon where conspecifics within a population of generalists exhibit differences in foraging behavior, each specializing on different prey types. Individual specialization is widespread in animals, yet is understudied in invertebrates, despite potential impacts to food web and population dynamics*. Sceliphron caementarium* (Hymenoptera: Sphecidae) is an excellent system to examine individual specialization. Females of these mud dauber wasps capture and paralyze spiders which they store in mud nests to provision their offspring. Individuals may make hundreds of prey choices in their short lifespan and fully intact prey items can be easily excavated from their mud nests, where each distinct nest cell represents a discrete foraging bout. Using data collected from a single population of *S. caementarium* (where all individuals had access to the same resources), we found evidence of strong individual specialization; individuals utilized different resources (with respect to prey taxa, prey ecological guild, and prey size) to provision their nests. The extent of individual specialization differed widely within the population with some females displaying extreme specialization (taking only prey from a single species) while others were generalists (taking prey from up to 6 spider families). We also found evidence of temporal consistency in individual specialization over multiple foraging events. We discuss these findings broadly in the context of search images, responses to changing prey availability, and intraspecific competition pressure.

## Lay Summary

Mud dauber wasp females hunt, sting, paralyze, and pack spider prey into a mud nest for their offspring to eat. Individual female wasps of the same species, in the same population, with access to the same resources have different preferences for the spider prey that they capture. Some females specialize on only one species of spider (prey specialists) while others catch a number of species (prey generalists). Individuals’ preferences remain consistent over time.

## INTRODUCTION

In the context of foraging, a species may be placed along a continuum from generalist to specialist depending on the breadth of their diet (Futuyma and Moreno 1988; Futuyma 2001). However, even within a single species of generalists, not all conspecifics are identical in their foraging behavior; some individuals in a population may specialize on one taxon (or a narrow range of taxa from those available), whereas other members of that population may specialize on different taxa (reviewed in Bolnick et al. 2003; Sargeant 2007; Araújo et al. 2011; Dall et al. 2012; Layman et al. 2015). This phenomenon is termed “individual specialization” (IS) and has been recorded in a number of animal species, though the proximate mechanisms driving it likely vary greatly (Araújo et al. 2011). For example, there may be genetic variation within a population that leads to innate individual differences in prey or host selection (Marchetti et al. 1998). Alternatively, an individual may learn how to effectively locate and handle a specific prey type and subsequently specialize on that prey type to increase its foraging efficiency or reproductive output (Otterbeck et al. 2015). The strength of individual specialization and how individuals partition resources in a population is largely driven by intraspecific competition and the resulting tradeoffs associated with using alternative resources (Svanback and Bolnick 2005).

The study of individual specialization has flourished in recent years with evidence from more than 189 animal species, yet its study remains limited by our ability to sample the diets of these groups (Araújo et al. 2011). For example, while sampling the scutes of turtle carapaces for stable isotope signatures or examining the gut contents of alligators can provide some sense of the nature of these animals’ diet, the information is still incomplete (Vander Zanden et al. 2013; Rosenblatt et al. 2015) and fails to provide further information about more detailed prey characteristics (such as the sex, age, and/or size of individual prey items) (Vander Zanden et al. 2013; Rosenblatt et al. 2015). Although recent reviews have called for a broader analysis of the temporal consistency of individual specialization over an animal’s lifetime (reviewed in Araújo et al. 2011; Layman *et al.* 2015); collecting such data in large, long-lived animals could take decades (e.g., Sargeant et al. 2005; Sargeant and Mann 2009; van de Pol et al. 2010; Woo et al. 2008; Vander Zanden et al. 2013; Patrick and Weimerskirch 2014; Kernaléguen et al. 2015; Rosenblatt 2015). Although studies on these organisms are immensely valuable, our ability to explicitly test hypotheses about the mechanisms that drive individual specialization in such species remains limited.

Mud dauber wasps (in the families Sphecidae and Crabronidae) provide a unique opportunity to study individual specialization in foraging behavior because all of the prey items that adult females collect remain paralyzed and intact within their mud nests until their offspring consume them (Shafer 1949). These wasps capture, sting, and paralyze spiders to feed their offspring (Coville 1987). Female mud daubers build mud nests and provision each cell of the nest with up to 25 live, paralyzed spiders before laying a single egg in the cell, sealing it up, and then moving on to build another nest cell (Shafer 1949). Thus, by sampling these mud nests, we have a complete catalog of every decision that a female made when foraging, such as the sex, age, size, and other features of individual prey items even without observing prey capture directly. The structure of the nest and the fact that each cell is sealed off before beginning to provision the next cell offers the unique ability to assess prey items in discrete foraging bouts. Adult female wasps only survive a single season (3–6 weeks) but take many prey items during this time in rapid succession (up to 25 spiders per day) (Shafer 1949). As such, we can examine the lifetime foraging decisions of individual females (potentially more than 400 foraging decisions) in a single summer which provides information on the temporal consistency in individual specialization. Finally, mud daubers build nests on human structures such as barns and bridges, often generating high densities that allow us to sample many nests from a single population where all individuals have access to the same resources. By sampling from a single population, where individuals are restricted to repeatedly returning to the same nest site, we can rule out other factors driving differences in individual foraging patterns (e.g., differential access to prey, different weather conditions, etc.).

Two previous studies have quantified individual foraging specialization from examination of field-collected mud nests in one group of spider-hunting wasps in the genus *Trypoxylon* (Araujo and Gonzaga 2007; Pitilin et al. 2012). These studies have provided a valuable starting point for using mud daubers as models to understand individual specialization. Here, we extend this work further into a separate mud dauber group and extend our analyses to examine the functional grouping (i.e., ecological guilds) of prey types, the temporal consistency of individual specialization, and the modularity and nestedness of the prey network.

The first goal of our study was to quantify individual specialization on spider prey within a single population of the black and yellow mud dauber (*Sceliphron caementarium).* By sampling freshly built nests and examining intact prey items, we first examined whether individual females were specializing differently on prey from particular taxa, ecological guilds, or size classes. Furthermore, we examined the degree of clustering in the niche network (i.e., modularity and nestedness) to further clarify how individuals’ foraging choices compared across the population. Such indices can help explain when and why diet specialization may occur in a population (Araújo et al. 2008; Tinker et al. 2012). We then measured temporal consistency in individual specialization, asking whether an individual’s foraging patterns remained consistent over multiple foraging bouts (by examining multiple adjacent cells of each individual female’s nest). Recent reviews have called for more attention to measures of consistency across multiple temporal scales of individual specialization (Layman et al. 2015); here we provide such data by examining foraging patterns over a multiday temporal scale in a short-lived insect.

## METHODS

### Study species

*S. caementarium* (Drury) is a common solitary wasp with a world-wide, cosmopolitan distribution (Krombein 1979) (Supplementary Figure S1a). Females build characteristic mud nests which are made up of cells, each containing one offspring (Supplementary Figure S1b). Females hunt, paralyze, and pack spider prey (up to 25 prey items) into a single cell (Obin 1992) (see Supplementary Figure S1c) before capping the cell and then moving on to construct the next adjacent cell (Shafer 1949). Populations of *S. caementarium* within a small geographic area range from extremely high density (i.e., thousands of nests densely packed under a single bridge) to fairly scarce (i.e., individual nests scattered singly on houses throughout a neighborhood) (E. Powell and L. Taylor, personal observation). Because these wasps hunt spiders exclusively, they might be particularly well-suited to helping us understand how levels of intraspecific competition (for limited prey) may affect patterns of individual specialization. *S. caementarium* prey has been well documented descriptively with proportions of spider prey taxa and average prey sizes provided for populations (Muma and Jeffers 1945; Horner and Klein 1979; Krombein 1979; Dean 1988; Volkova et al. 1999; Polidori et al. 2007) but never quantified at an individual level. *S. caementarium* has been successfully used in prey choice tests in captivity (Blackledge and Pickett 2000; Blackledge et al. 2003; Uma and Weiss 2010; Uma et al. 2013); however, no study has looked at individual differences in behavior (i.e., individual specialization) in either captive or free-ranging *S. caementarium*.

### Nest collection and prey classification

We collected 91 nest cells from the nests of 30 females from a single, dense population under a bridge over Otter Creek, in north central Florida, USA in May and July 2015 (Supplementary Figure S1d). We considered each nest to be built by an independent female because females construct multiple cells on the same nest over time (Shafer 1949). Additionally, similar stages of larval development in each adjacent cell suggested that females had not diverged from a single nest during the collection period. The following criteria were used to locate nests for data collection: 1) nests had to be freshly built (meaning they were made during the season they were collected and contained freshly paralyzed, live spiders), 2) they had at least one sealed cell with no evidence of parasitism, and 3) they were accessible for collection with a step ladder (e.g., not over the body of water under the bridge). Nest cells that had less than 5 intact spiders remaining were not included in our study. After excluding individual cells with less than 5 intact spiders, we randomly selected up to 3 cells from each female’s nest, resulting in a total of 796 prey spiders for our analyses. We chose to randomly select only 3 cells from each nest because at the time of sampling, most nests in our population had between 1 and 3 cells completed (with a maximum nest size of 7 cells). Because these wasps build their nest cells in clumps (see Supplementary Figure S1b), it is impossible to confidently determine the order in which the individual cells were built.

Spiders were identified to the genus level using Ubick et al. (2005) with updated family classification used for the families Nephilidae and Eutichuridae (World Spider Catalog 2016). We classified spiders by taxonomic family initially because *S. caementarium* as a species is known to take a wide variety of spider families with up to 14 families documented (Muma and Jeffers 1945; Horner and Klein 1978; Dean 1988; Volkova et al. 1999); this is in contrast to other mud dauber wasps where species specialize on only one spider family (e.g., *Trypoxylon albonigrum* on Araneidae and *Trypoxylon agamemnon* on Anyphaenidae) (Araujo et al. 2007; Pitilin et al. 2012). Many families of spiders represented in our prey sample are large and diverse (e.g., Araneidae with 169 genera and Salticidae with 598 genera) (World Spider Catalog 2016). For this reason, we went on to classify spiders at the genus level to more closely examine patterns of individual specialization that may have been missed by focusing only on the family level. Specifically, genera within diverse taxonomic families often use different defensive strategies to avoid predation (Blackledge 1998; Blackledge and Wenzel 1999), structure vastly different webs (Stowe 1986; Foelix 2011), orient differently within the web (Zschokke and Nakata 2010), and differ in aggregation behavior (Foelix 2011); as such, we might expect individual females to be most likely to specialize at the genus level. A small number of spiders in our sample (*n* = 6) were damaged due to feeding by the wasp larvae and were not identifiable to genus; these spiders were excluded from the genus-level analyses.

In addition to taxonomic classification, we also grouped spiders by ecological guild following the recent guild categories established for spiders by Cardoso et al. (2011). Recent studies in other animals have shown that grouping prey items into functional groups (rather than taxonomic groups) can be more useful in quantifying patterns of individual specialization (e.g., Newsome et al. 2015). Handling strategies or habitat may be consistent over multiple taxonomic classifications; if an animal is learning a strategy or utilizes a particular habitat for foraging, then patterns of individual specialization will be clearer when prey are grouped into ecological guilds (Hatase et al. 2007; Fodrie et al. 2015; Newsome et al. 2015). In *S. caementarium*, females may learn life-history attributes (specific to an ecological guild) of their spider prey, such as their habitat, web type, defense mechanisms, or other behavior (Pitilin 2012); as such, classifying prey by ecological guild may be an informative way to define spider prey groups. We also considered examining individual specialization on prey sex for similar reasons, as male and female spiders often differ in habitats and behavior (Aisenberg et al. 2007). However, immature spiders (that were unable to be sexed) made up 69.8% of our sample; while incorporating prey sex into future studies of individual specialization may be informative (in cases where the prey sample is not dominated by immature prey), we did not include sex in the present analyses.

Because the *S. caementarium* in our study took a wide range of prey sizes (total body length range: 2.95 mm–14.7 mm, carapace width range: 0.9 mm–3.7 mm), we also considered the possibility that individuals were specializing specifically on prey size (i.e., consistently taking prey of the same size, regardless of taxa). Other wasps, including at least 2 spider-hunting mud daubers and a grasshopper-hunting wasp, are known to individually specialize on prey of the same size (Araujo et al. 2007; Pitilin et al. 2011; Santoro et al. 2011). For all prey items taken, we took 2 measurements: the total body length (tip of the carapace to the tip of the abdomen) and carapace width (at its widest part) to the nearest hundredth of a millimeter. We chose these measurements as an alternative to measuring dry weights which would have destroyed the specimens. Spiders that had been damaged by feeding wasp larva (*n* = 24) were not included in size analyses. All spiders sampled are preserved in 70% ethanol and have been deposited at the Florida State Collection of Arthropods in Gainesville, FL.

### Statistical analyses

To determine whether *S. caementarium* in our study population were individually specializing with respect to the taxonomic group (family or genus) or ecological guild of spider prey taken, we first randomly selected one nest cell from each female in the population (*n* = 30, containing between 5 and 22 spiders per cell) and used the PSicalc function in the R package RInSp to quantify levels of individual specialization (Zaccarelli et al. 2013). RInSp calculates a measure of individual specialization proposed by Bolnick et al. (2002); specifically, proportion similarity indices (PS_i_ values) are calculated for each individual, which describe the overlap between that individual’s diet and the diet of the entire population. The PS_i_ values range from 0 to 1, with specialists (that overlap little with the rest of the population) falling near 0 and generalists (that overlap widely with the rest of the population) falling close to 1. The level of individual specialization for the entire population (Index of Similarity, or IS) is then calculated as the mean of the individual PS_i_ values. A Monte Carlo resampling procedure (with 999 replicates) is then used to calculate a *P*-value to test the null hypothesis that all individuals are sampling equally from the overall distribution of spider prey taken.

For each of the above analyses, we used RInSp to calculate the clustering coefficient, C_ws_, following Araújo et al. (2008), which helps us understand the modularity of the niche network (Zaccarelli et al. 2013). Specifically, a C_ws_ value close to 0 indicates no modularity; this could occur if either 1) the population is made up entirely of generalists or 2) that specialists and generalists coexist in the population and specialist diets are nested within those of the generalists (Kernaléguen et al. 2015). If the C_ws_ value is less than 0 (closer to −1), the network is made up of separate, discrete groups of specialists where each group forages on a specific resource or set of resources. If the C_ws_ value is greater than 0 (closer to 1), there are not discrete groups; instead, specialist individuals all exhibit relatively unique diets (Zaccarelli et al. 2013). A Monte Carlo resampling procedure (with 999 replicates) was used to calculate a *P*-value to test the null hypothesis that variation in diet was a result of individuals choosing at random from the available resources (Zaccarelli et al. 2013).

Because we found that our networks showed very little modularity (as indicated by C_ws_ values near 0, see Results for details), we went on to test for the strength of nestedness within the network (for each of our analyses described above). Theory indicates that such indices may give insight to the ecological causes (e.g., intraspecific competition pressures) of diet partitioning in a population (Araújo et al. 2008; Tinker et al. 2012). Using ANINHADO 3.0 software (Guimaraes and Guimaraes 2006), we calculated the nestedness index NODF (an acronym for nestedness based on overlap and decreasing fill; see Almeida-Neto et al. 2008). To assess the significance of our nestedness values, we compared them with values produced from null models (999 replicates, using null model 2 following Bascompte et al. 2003). The NODF index ranges from 0 to 100 where 0 indicates an absolute absence of nestedness and 100 indicates complete nestedness.

Because the above procedures are suited only for the analysis for discrete variables, we used the WTcMC function in RInSp to quantify levels of individual specialization on the continuous variable of prey size (both body length and carapace width) (Zaccarelli et al. 2013). This function calculates the total niche width of the population (TNW), which is broken down into between-individual and within-individual components (BIC and WIC, respectively) following Roughgarden (1974). The degree of individual specialization (IS) is calculated as WIC/TNW; as above, values of IS fall between 0 and 1, with smaller values indicating higher levels of individual specialization. A Monte Carlo resampling procedure is then used to calculate a *P*-value to test the null hypothesis that all individuals are sampling equally from the overall distribution of spider prey sizes taken.

Our analyses described above suggested that individuals were specializing both with respect to prey taxa and as well as with respect to prey size (see Results for details); because different prey taxa might be different sizes (i.e., some families or genera might be larger than others), these analyses alone don’t allow us to disentangle whether taxa or size is the major driver of the patterns of IS observed. For example, if individuals are specializing on prey taxa that are different sizes, then apparent individual specialization on prey size might simply be a byproduct of individual specialization on prey taxa. To examine this possibility, we compared the mean body sizes (body length and carapace width) of the 3 most common families in our sample (representing 94% of the population sample) using an Anova. If there are no size differences between the taxa, we can conclude that females are indeed specializing mainly with respect to size, regardless of taxa. Alternatively, if there are differences in size among taxa, then either size or taxa could be driving female foraging choice.

Another way to tease apart IS on prey taxa versus prey size is to focus on a subset of individuals in the population that are feeding exclusively on one particular taxa and examine IS with respect to prey size in this group. The nests of 9 individuals in our population, collected on the same day, foraged exclusively on *Nephila clavipes* (carapace width range: 0.98–3.7 mm; total body length range: 4.3–14.7 mm). We calculated the prevalence of IS on size within this group.

To examine whether patterns of individual specialization remained consistent over time, we compared levels of IS calculated at 2 temporal scales for subset of the females (*n* = 14) for which we had 3 intact nest cells. Using RInSp (Zaccarelli et al. 2013), we first calculated PS_i_ values for each individual female using data from a single randomly selected nest cell (as described in the above analyses) and compared these values with PS_i_ values calculated for the same females using the combined data from 3 randomly selected cells from each nest. Each cell in this sample represents a foraging bout that includes between 5 and 18 prey items (likely collected in rapid succession in a single day) (Shafer 1949; Coville 1987), so this allowed us to examine if and how patterns of specialization drifted over a period of several days. If wasps are consistent within a single foraging bout but drift in what they specialize on from one day to the next, we would expect mean PS_i_ values from the single randomly selected cell to be lower (closer to 0 and more specialized) compared with the mean PS_i_ values based on data from 3 nest cells. Alternatively, if patterns of IS are consistent over several days, we would expect either no difference between these 2 measures of IS, or higher mean PS_i_ values for the single cell data (compared with the 3-cell data). We compared the mean PS_i_ values using paired *t*-tests in JMP version 12 (SAS Institute, Cary, NC).

## RESULTS

**Figure 1:**
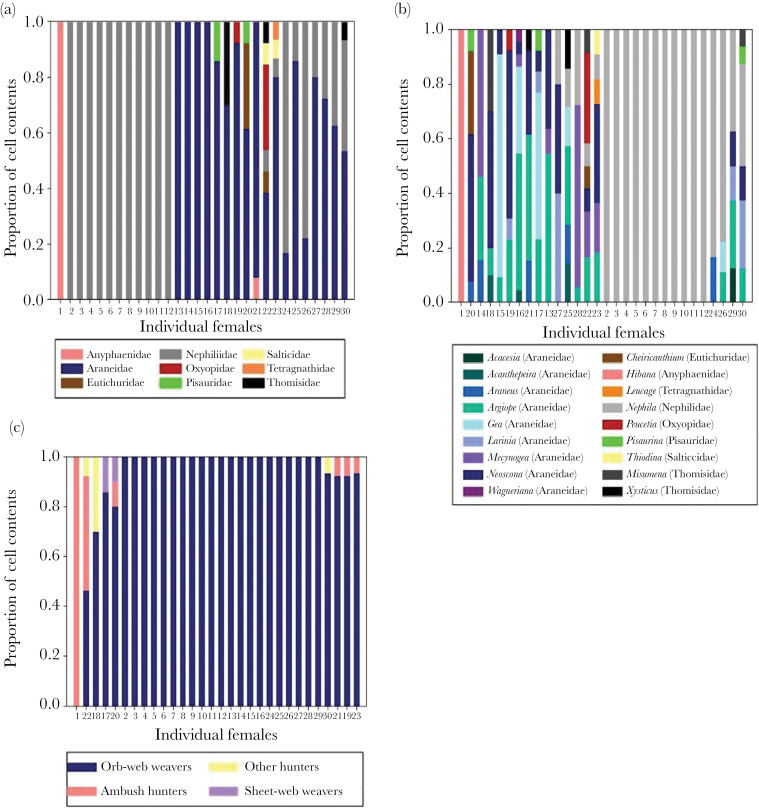
Proportions of spider prey types in randomly selected nest cells of individual female *Sceliphron caementarium.* Subtle patterns of specialization differ depending on whether prey are classified by taxonomic family (a), genus, (b), or ecological guild (c). Within each graph, individual females are ordered by their PS_i_ values, with lowest PS_i_ values (more specialized individuals) on the left and highest PS_i_ values (prey generalists) on the right. Individuals have unique ID numbers so that they can be compared across graphs. These figures illustrate how specializing on a single prey type does not necessarily equate to having the lowest PS_i_ value (e.g., see individuals 2–12). Instead, the lowest PS_i_ values are found in females that focus on a particular prey type that is not used by other females in the population (e.g., see individual 1). Note: differences between prey categories are best viewed in the color version of this figure available online (open-access).

Individual female *S. caementarium* foraged on different prey taxa from one another. We found evidence of individual specialization with respect to both prey family and prey genus; in both cases, there was a large range between individual PS_i_ values, suggesting that some individuals (with low PS_i_ values close to 0) were acting as prey specialists while others (with high PS_i_ values close to 1) were acting as prey generalists (Figure 1a and b, Table 1). Some females in the population specialized exclusively on one species of spider, whereas another female took spiders from 7 genera (spanning 6 spider families) (Figure 1a and b). The lowest PS_i_ values indicate individuals who not only favored one particular prey type but who chose a prey type that was otherwise uncommon in the nests of other females (Figure 1a and b).

**Table 1:** Results of tests for individual specialization based on contents of field-collected nests of *Sceliphron caementarium*

Level of prey classification	Range of individual PS_i_ values	Population IS value	*P*
Family	0.04–0.88	0.51	0.001*
Genus	0.04–0.67	0.39	0.001*
Ecological guild	0.07–0.98	0.87	0.001*

*P* values indicating significant individual specialization are indicated with an asterisk (*). PS_i_ values (Proportion Similarity indices) are calculated for each individual, whereas IS values (Indices of Similarity) are calculated for the entire population. In both cases, values close to 0 indicate prey specialization, whereas values close to 1 indicate prey generalization.

For the analyses of prey taxa, the clustering coefficient of the population was close to 0 (family-level analysis: C_ws_ = 0.09, *P* < 0.001; genus-level analysis: C_ws_ = 0.08, *P* = 0.009). Because our individual PS_i_ values indicated that there were both specialists and generalists in the population (see Table 1), this low value of C_ws_ suggests that the diets of specialist individuals were nested within the diets of generalist individuals (i.e., specialist diets were made up of smaller subsets of the prey taken by generalists) (e.g., Kernaléguen et al. 2015). Our NODF index also indicates significant nestedness in the network (see Table 2).

**Table 2:** Indices of nestedness (NODF) calculated for the *Sceliphron caementarium* individual-resource network across 3 different classification levels for prey

Level of prey classification	NODF	NODF_null_	*P*
Family	21.20	13.28	0.02*
Genus	36.08	23.79	<0.001*
Ecological guild	18.07	18.50	0.53

*P* values indicating significant nestedness are indicated with an asterisk (*). NODF values range from 0 to 100 where 0 indicates an absence of nestedness and 100 indicates complete nestedness. Null model NODF values (as described in the text) are presented here for comparison.

Female wasps also individually specialized on the ecological guild of their spider prey (Figure 1c, Table 1). Again, PS_i_ values varied widely across the population but most individuals foraged solely on orb weaving-spiders (comprising 91.17% of the prey taken). Surprisingly, 2 females foraged across 3 different ecological guilds, where spiders differed substantially in their life-history and habitat type (Figure 1c). As in the genus- and family-level analyses, the clustering coefficient was close to 0 (C_ws_ = 0.01, *P* < 0.001), again suggesting that specialist individuals were nested within the diets of generalists (e.g., Kernaléguen et al. 2015). However, our NODF values of nestedness in this analysis did not reveal significant degrees of nestedness.

**Figure 2:**
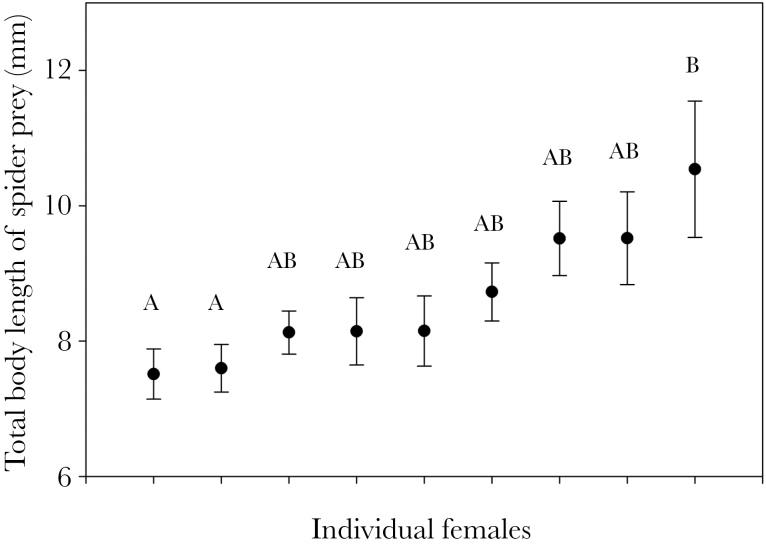
Variation in body length of prey captured by individual *Sceliphron caementarium* that were specializing exclusively on the golden orb-weaving spider, *Nephila clavipes* (mean ± SEM). Some females differed from others in the mean total body size of prey items. Different letters indicate significantly different prey sizes among individual females.

Individuals consistently foraged on differently sized prey items with respect to both carapace width and total body length of the spider prey (WIC/TNW = 0.75, *P* = 0.001, *N* = 30; WIC/TNW = 0.58, *P* = 0.001, *N* = 30). However, because the 3 most common spider families significantly differed in size (carapace width: *F*_2, 327_ = 6.48, *P* = 0.0017; total body length: *F*_2, 308_ = 46.04, *P* < 0.001), it is difficult to determine whether prey taxa or prey size is the major driver of individual specialization. Among wasps that foraged exclusively on the golden orb-weaving spider, *N. clavipes* (whose nests were collected on a single day) some females differed significantly in total body length of their prey (*F*_8, 93_ = 3.16, *P* > 0.003, *n* = 9; Figure 2). With this subset of females, we still found significant individual specialization on prey size (carapace width: WIC/TNW= 0.81, *P* = 0.03, *N* = 9; total body length: WIC/TNW = 0.74, *P* = 0.003, *N* = 9).

Examining data across 2 temporal scales (i.e., comparing data from a single foraging bout to data from 3 foraging bouts), we found that individual females remained consistent in their specialization. For the family-level analyses, PS_i_ values did not differ between 1 and 3 foraging bouts (*t*_13_ = 1.93, *P* = 0.075). In the genus-level analyses, PS_i_ values decreased with 3 foraging bouts (compared to data from a single foraging bout) (*t*_13_ = −4.89, *P* = 0.0003).

## DISCUSSION

Within a single population of *S. caementarium*, we found that individual females consistently foraged on different types of spider prey. We found strong patterns of individual specialization with respect to taxonomic family, genus, ecological guild, and the size of prey items taken. Interestingly, some individuals in the population were prey specialists that took only a single species of spider prey or focused on prey species that were uncommon in the nests of other females. In contrast, there were also prey generalists who took a wide variety of prey types within a single foraging bout (spanning 6 families and 7 genera in a single nest cell). Our data also reveal significant patterns of nestedness, meaning that the prey in a specialist’s diet was typically a subset of the prey taken by more generalist females. In previous studies across animal taxa, patterns of individual specialization become weaker over time, as an individual’s prey preferences drift or as seasonal differences alter prey composition (Fodrie et al. 2015; Novak and Tinker 2015; Kernaléguen et al. 2016). However, the degree of individual specialization that we observed in our study was consistent across multiple foraging bouts. This study lays the groundwork for future studies to examine the mechanisms and implications of individual specialization in these mud dauber wasps.

Although direct comparisons between the analyses of IS at different levels of prey classification (i.e., genus, family, and ecological guild) are impossible due to the differing number of resources within each analysis, we found the highest degree of individual specialization (as well as the highest degree of nestedness) when we analyzed data at the genus level (see Tables 1 and 2). This suggests that individual wasps may be developing search images or learning hunting strategies that are uniquely suited to specific genera of spider prey. This is not surprising, as closely related spider genera often differ quite dramatically in their morphology and behavior (Ubick et al. 2005). For instance, wasps in our study captured prey from 9 different genera within the large and diverse spider family Araneidae. These araneid genera have vastly different defensive strategies such as the web-shaking behavior of *Argiope* that presumably deters predators (Tolbert 1975) and the hard spines of *Acanthepeira* that might make handling and consumption difficult (Ubick *et al.* 2005). Moreover, several genera also differ in aspects of morphology that would likely affect a foraging wasp’s search image, such as the conspicuous body coloration of *Mecynogea* (Bradley 2013) or the web-decorations (stabilimenta) common in *Argiope* (Herberstein 2000). Furthermore, some araneid genera are diurnal, spending their time in the center of their web during the day (e.g., *Argiope*), while others are nocturnal, spending their days hiding in a silken retreat or cryptically perching against tree bark (e.g., *Neoscona*) (Chuang et al. 2008). In contrast to the genus-level analyses, we saw much lower levels of IS (and nestedness) in our guild-level analyses (see Tables 1 and 2). This may be because these guild classifications are based primarily on spider hunting strategies (e.g., orb weaving, ambush hunting, etc.; see Cardoso et al. 2011) rather than specific defensive strategies that are more likely to be relevant to a spider-hunting wasp. Future work is clearly needed to examine how individual wasps respond to, and potentially learn from, such variation in spider prey using close observations of wasp-spider interactions.

The strong individual specialization that we documented in *S. caementarium* is likely a consequence of their unique life history. A 2011 survey of 142 studies of individual specialization (across various animal taxa) found a mean IS value of 0.47 (Araújo et al. 2011); this is comparable to the degree of individual specialization observed in our study (see Table 1). Our study documents the third mud dauber wasp to individually specialize on spider prey (see also Araujo and Gonzaga 2007; Pitilin et al. 2012). Individual specialization on spider taxa in mud dauber wasps may be driven by high levels of intraspecific competition, as females often build nests in close proximity to others within relatively constrained nesting sites (e.g., under bridges or other structures) (Araújo et al. 2011). Indeed, our focal population consisted of thousands of mature adult females actively building nests under a single bridge (see Supplementary Figure S1d). As these wasps exclusively hunt spiders, each individual taking up to 25 spiders per day over the entire summer (Obin 1992), competition for spiders is likely to be intense. Moreover, fitness is likely tied directly to foraging efficiency; the faster a female can collect spiders, the more nest cells and offspring she can provision. Furthermore, parasites and parasitoids plague mud dauber wasp nests and faster foraging likely allows females to provision and seal the nest as quickly as possible (Araujo and Gonzaga 2007; Pitilin et al. 2012). Although the high densities of *S. caementarium* in our focal population are not unusual, there are certainly comparable habitats that are more sparsely populated (E. Powell and L. Taylor, personal observation); an interesting next step would be to examine levels of IS along a gradient of population density to assess how competition might shape patterns of IS.

One of our most interesting findings that has not been reported in previous studies of mud dauber wasps is that specialists and generalists coexist within the population and that specialists’ diets were nested within those of the generalists. Females varied from taking exclusively one species of spider to capturing prey items across 6 families from different ecological guilds, despite the fact that all females presumably had access to the same resources. In fact, one individual in the population exhibited extreme individual specialization (family level and genus level PS_i_ = 0.04), foraging almost exclusively on the genus *Hibana* (Family: Anyphaneidae) with the exception of 2 juvenile spiders in the family Pisauridae. This female captured 61 *Hibana* spiders (including males, females, and juveniles) to provision 5 nest cells, consistently specializing on a resource that other females in the population ignored (only a single additional *Hibana* female was found in any other sampled nest). Despite this extraordinary individual female, we found significant patterns of nestedness in the overall population (see Table 2).

Different models of diet specialization are expected to lead to different patterns of nestedness (Svanback and Bolnick 2005) and our findings allow us to weigh support for such competing models. According to the “shared preferences” model, proposed by Svanback and Bolnick (2005), a nested network should emerge under high intraspecific competition (Svanback and Bolnick 2005; Araújo et al. 2008; Araujo et al. 2010). The “shared preferences” model posits that all individuals have a preferred prey type but will add novel prey types to their diet as competition increases; thus, the diets of more choosy individuals (specialists) are nested subsets of less choosy individuals (generalists)(Svanback and Bolnick 2005). Because the preferred prey type presumably has the lowest handling time (due to past experience and learning), individuals should stick with this resource when possible (Svanback and Bolnick 2005). In contrast, under the “competitive refuge” model, individuals are not expected to exhibit nested diets; instead, when competition increases individuals prefer different second-choice resources from one another (Svanback and Bolnick 2005). A third model, “distinct preferences”, postulates that little nestedness occurs even at high levels of competition because discrete specialist phenotypes prefer different first-choice resources (Svanback and Bolnick 2005). In our mud dauber population, the high densities and expected high intraspecific competition, paired with nested diets of our focal population, are consistent with the “shared preferences” model (see Supplementary Figure S1d); again, more work should be done to examine how patterns of nestedness differ across gradients of population density. We expect that such individual variation in prey preference (where diets of more choosy individuals are nested within the diets of less choosy individuals) within our wasp population may result from the learning of search images (visual, olfactory, or both). If these search images are formed on a female’s first foraging flight, then they may be influenced largely by chance (depending on which prey item the spider first encounters).

Our results suggest a population in which both generalist and specialist strategies coexist because they confer similarly high fitness in certain situations. Specialists likely develop a search image for one prey type and become extremely proficient in the finding and handling of that prey type, thereby increasing their foraging efficienc (Otterbeck et al. 2015). Such a specialist strategy could be successful unless that one resource disappears or competition increases (e.g., Wolf and Weissing 2012). In contrast, generalists are more likely to forage on multiple novel prey types, which may reduce their foraging efficiency on any one prey type but would be advantageous in times of high competition or during shifts in resource availability. Similar instances of specialist and generalist strategies co-occurring have been reported in other systems: For example, in the bluegill sunfish, habitat specialists were more successful foragers than habitat generalists but specialists neglected to utilize new resources during environmental fluctuation (Werner et al. 1981). In our system, we found the degree of generalization by some individuals to be particularly striking. For instance, one individual was able to capture prey from up to 6 spider families to provision a single nest cell, presumably in a single day (see Figure 2a). The range of spider families that she captured included web builders (e.g., Araneidae and Nephilidae), sit-and-wait ambush hunters (e.g., Thomisidae and Oxyopidae), and active visual predators (e.g., Salticidae). Given how different the members of these spider families are, it is difficult to imagine how a single female mud dauber would be able to successfully capture such a wide variety of prey types in such a short period of time. An explanation for this may come from an interesting natural history observation. On at least one occasion, we observed an individual female stealing spider prey from another female’s nest (before the nest cell was capped with mud). It may be that the prey “generalists” that our study revealed are actually kleptoparasitic “specialists,” that steal this assortment of prey from several other individuals. Given the high density of wasps at our field site, this could certainly be a viable strategy but has not previously been reported in the literature. This possibility should be examined further with careful observations of individually marked wasps in the field.

In an analysis of a subset of 9 females that foraged exclusively on the golden orb-weaving spider, *N. clavipes*, on the same day, individuals also specialized on prey size (with some individuals favoring large *N. clavipes* and some individuals favoring smaller *N. clavipes*). Similar patterns of IS on both taxa and size have been found in 2 previous studies of mud dauber wasps (Araujo and Gonzaga 2007; Pitilin et al. 2012), suggesting that this may be a widespread pattern in mud daubers. Because these wasps need to fly with these prey items back to their nests, we might expect larger individuals to favor larger prey items (e.g., Santoro et al. 2011), yet this pattern remains to be tested in *S. caementarium*.

Another intriguing finding that differs from most studies of individual specialization in other taxa is that individual females in our study remained relatively consistent in their prey choices over time. When our data were pooled across multiple foraging bouts, we found that female PS_i_ values either remained the same or increased, suggesting consistency in individual specialization over time. It seems plausible that a female’s search image may even strengthen with experience; if this were the case, we might expect her to become more consistent in her prey choices over time. Because we are unable to determine the exact order of provisioning of the nest cells in the present study, we are unable to examine this possibility here. Future work should explore this possibility in more detail. In most other studies of IS, only a small portion of the reproductive lifespan of a predator or a small window of prey availability can be examined. It has been argued that this may result in artificial increases in the level of individual specialization caused by an underrepresentation of the entire niche width available over multiple seasons (Araujo and Gonzaga 2007; Novak and Tinker 2015; Kernaléguen 2016). However, short-lived mud daubers have access to only a single season of prey items in their lifetime (Shafer 1949). By examining 2 temporal scales and finding that IS remains consistent, our data suggest that a short sample period does not artificially inflate IS values in *S. caementarium*.

Overall, our data reveal intriguing patterns of individual specialization in an understudied system that warrants further investigation. For example, *S. caementarium* is an ideal system to evaluate how individual specialist predators respond to changes in resource availability, such as the introduction of invasive prey species, and the flexibility of search images with new and changing resources. With an expansive cosmopolitan distribution, *S. caementarium* provides the opportunity to examine patterns of individual specialization in areas with extreme variation in available prey types. In our study population in Florida, *N. clavipes* was the most common prey item sampled (making up 73.37% of the population’s prey with 9 of 30 females specializing exclusively on *N. clavipes*). In northeastern Georgia, an Asian congener, *Nephila clavata* (the Joro spider), was recently introduced (Hoebeke et al. 2015) and we have already found these spiders in nests of *S. caementarium* in the area (Powell E, Matthews R, Hoebeke R, and Taylor L, unpublished data from Jackson County, GA). We propose that mud daubers could be used not only to track and monitor this newly introduced species as it spreads from its introduction site, but also to understand how individual specialization may shape the way that a population responds to introductions of exotic prey; such ideas are rarely explored in applied contexts (see review by Araújo et al. 2011). Similarly, although generalist species are often overlooked as biological control agents (Louda et al. 2003), it is becoming clearer in many species that generalist populations are made up of specialist individuals (Bolnick et al. 2003; Araujo et al. 2007). The rich and growing literature on individual specialization likely has a lot to contribute to both our understanding of basic ecology as well as more applied fields like agriculture and conservation. Mud daubers, in particular, seem especially well-suited for such research directions.

## SUPPLEMENTARY MATERIAL

Supplementary data are available at *Behavioral Ecology* online.

## FUNDING

This work was supported by funding from a National Science Foundation grant (IOS-1557867 to L.A.T.), the Florida Museum of Natural History, and the Entomology and Nematology Department at the University of Florida. Publication of this article was funded in part by the University of Florida Open Access Publishing Fund.

## Supplementary Material

PowellTaylor_IS_Figure_S1_3March_EPedit
