# Supplementary material for: Specialists and generalists coexist within a population of spider-hunting mud dauber wasps
Source: Behav Ecol. 2017 Apr 1;28(3):890–8. doi: 10.1093/beheco/arx050 (PMC5873241; doi:10.1093/beheco/arx050)
Supplement: PowellTaylor_IS_Figure_S1_3March_EPedit [file arx050_suppl_powelltaylor_is_figure_s1_3march_epedit.docx]

Supplementary Material

Powell EC and Taylor LA

Specialists and generalists coexist within a population of spider-hunting mud dauber wasps

*Behavioral Ecology*

**
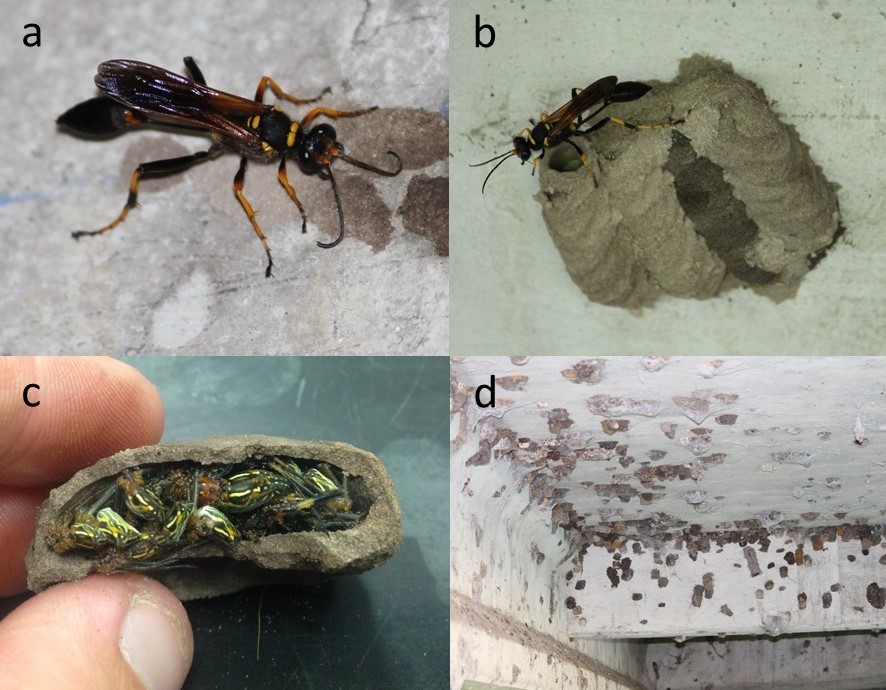
**

**Figure S1.** (a) Adult female *Sceliphron caementarium,* (b) *S. caementarium* nest with an adult female resting on the surface, (c) cross section of a single nest cell packed with paralyzed spider prey, (d) a small section of our focal population with high densities of *S. caementarium* nests.
